# Estimation of Steering and Throttle Angles of a Motorized Mobility Scooter with Inertial Measurement Units for Continuous Quantification of Driving Operation

**DOI:** 10.3390/s22093161

**Published:** 2022-04-20

**Authors:** Jun Suzurikawa, Shunsuke Kurokawa, Haruki Sugiyama, Kazunori Hase

**Affiliations:** 1 Department of Assistive Technology, Research Institute, National Rehabilitation Center for Persons with Disabilities, 4-1 Namiki, Tokorozawa-shi 359-8555, Japan; black.shun.0822@gmail.com (S.K.); sugiyama-haruki@ed.tmu.ac.jp (H.S.); 2 Department of Mechanical Systems Engineering, Graduate School of Systems Design, Tokyo Metropolitan University, 1-1 Minami-Osawa, Hachioji-shi 192-0397, Japan; kazunori.hase@tmu.ac.jp

**Keywords:** driving recorder, skills evaluation, assistive technology, powered mobility device, acceleration

## Abstract

With the growing demand from elderly persons for alternative mobility solutions, motorized mobility scooters (MMSs) have been gaining importance as an essential assistive technology to aid independent living in local communities. The increased use of MMSs, however, has raised safety issues during driving and magnified the necessity to evaluate and improve user driving skills. This study is intended to develop a novel quantitative monitoring method for MMS driving operation using inertial measurement units (IMUs). The proposed method used coordinate transformations around the rotational axes of the steering wheel and the throttle lever to estimate the steering and throttle operating angles based on gravitational accelerations measured by IMUs. Consequently, these operating angles can be monitored simply using an IMU attached to the throttle lever. Validation experiments with a test MMS in the stationary state confirmed the consistency of the proposed coordinate transformation with the MMS’s geometrical structure. The driving test also demonstrated that the operating angles were estimated correctly on various terrains and that the effects of terrain inclination were compensated using an additional IMU attached to the scooter body. This method will be applicable to the quantitative monitoring of driving behavior and act as a complementary tool for the existing skills’ evaluation methods.

## 1. Introduction

A motorized mobility scooter (MMS) is an important assistive technology for elderly persons with reduced movement functions in their lower limbs [1,2,3]. Previous studies have indicated the positive effects of MMSs on the autonomic social participation of users in local communities because of their improved freedom of mobility [4,5]. The MMS can be used as a replacement for walking or use of a car for outside activities involving various travel distance ranges, including a simple stroll, shopping, and participation in family events [6]. User satisfaction with MMSs is reported to be high, as is the frequency of MMS use, which ranges from daily to several times per week for the majority of users [7]. The growing roles of MMSs have motivated researchers in related fields to evaluate and enhance the outcomes of MMS use by various means. However, these efforts are far beyond those being made for other conventional powered mobility devices (PMDs), such as the powered wheelchair (PWC) [8].

PMDs that are intended for elderly persons or persons with disabilities commonly have an issue to be overcome, e.g., safety when driving [9,10,11,12,13,14]. Unlike automobiles, neither a license nor official legally regulated training are required to drive PMDs [13,15]. Despite this ease with which usage can begin, the standard users of MMSs have often suffered considerable decline in their sensory and cognitive functions, in addition to movement functions [16,17]. While PWCs are usually prescribed by medical professionals based on a careful assessment of the individual’s body functions [18], MMSs are likely to be acquired personally without any consultations with professionals [19,20]. Interview-based studies of MMS users showed that these users have concerns over safety issues, and the quality and quantity of the training that they each received on acquisition of an MMS differed greatly between individuals [21,22]. Carlsson and Lundälv revealed that the number of accidents relating to MMSs in Sweden increased by three times over the period from 2007 to 2016 [23].

Although there is a strong demand for safety assurance when driving an MMS, few solutions have been implemented to date. Previous studies have proposed the use of robotics-based methods, including environmental sensing and vehicle control, to improve the safety of MMSs [24,25,26]. For example, Eck and colleagues developed a drive assistance system for a MMS that consisted of velocity and steering controllers, a collision avoidance system, and an navigation system that used the Global Positioning System [24]. Similar types of assistance systems have also been developed by other groups using various sensing and information processing technologies [25,26]. However, these systems have not been used in practice to date. According to the literature, the cost of this additional apparatus appears likely to be the primary reason for the slow spread of these technology-oriented solutions [27].

Another important and more cost-effective solution is to evaluate and improve the driving skills of individual MMS users. Several MMS user training protocols have been developed, trialed, and proven to be effective in improving user driving skills [28,29,30]. To confirm the effects of such training, the measurement of the user’s driving skills is as crucial as the training. Driving skills are generally evaluated using validated measures for MMSs, most of which are analogous to the measures for PWCs [31,32,33,34]. Since these measures are based on the manual observation of driving tasks, the use of the measures requires trained raters. Even when the raters are highly experienced, however, the use of visual observation means that they may possibly overlook some substantial characteristics of the driving operations and scooter behaviors, resulting in a lack of quantitative assessment and/or characterization of the changes in the user’s driving skills. 

Recent studies on PWC driving have revealed that the measurement and analysis of the joystick inputs provide plentiful insights into the interactions between the driver and the wheelchair. Suzurikawa and colleagues quantified the operational burden using the joystick input signals to evaluate the effect of a downhill-turning prevention control that was implemented on a PWC [35]. Sorrento and colleagues also analyzed the joystick signals of PWCs during driving and found differences between the joystick operation patterns of novices and those of experienced PWC users [36]. While these studies used specially modified joystick units to record the input signals, Rabreau and colleagues proposed an inertial measurement unit (IMU)-based logger of joystick movements that could easily be plugged into the tip of the joystick [37]. 

Despite these pioneering efforts for PWCs, there have been few studies involving quantitative measurements and evaluations of MMS operations. The fact that the interface configuration of the MMS is quite different from that of a PWC may hamper the measurement of MMS operations. While a PWC has a two-degrees-of-freedom (2-DOF) joystick as its operating interface, an MMS has two 1-DOF interfaces, i.e., a throttle lever used for speed control and a steering tiller for directional control [13]. The inputs to the PWC joystick and the throttle lever angle of the MMS are electrically monitored and are converted into the angular velocities for the motorized wheels. Meanwhile, the steering tiller of the MMS is connected to the front wheel(s) mechanically and thus modulates the driving direction directly. Previous studies of an MMS simulator did not mention a quantitative analysis of the operation, either, although these studies may have measured the driving operations during the performance of the tasks for use as the control parameters for the simulations [38,39,40].

This study aims to develop a continuous monitoring system for MMS driving operations that can be installed easily and without electrical modification of the scooter body. To achieve this objective, we applied an IMU that can be used by simply attaching it to the operating interface of the MMS and then detecting the changes in the acceleration caused by the driving operation. An equation with rotation matrices that represents the geometrical relationship between the IMU and the two rotational axes of the operating interfaces can be solved to estimate the throttle and steering angles. Previous studies on IMU-based estimations of human motion and joint angles during ambulation also used a similar calculation approach and reported various sources of estimation errors, including vibration and misalignment of the IMU axes [41,42,43,44]. We also evaluated the estimation accuracy of the proposed method by implementing the IMU system on a test MMS. 

## 2. Materials and Methods

### 2.1. Structure of the Test MMS 

**Figure 1:**
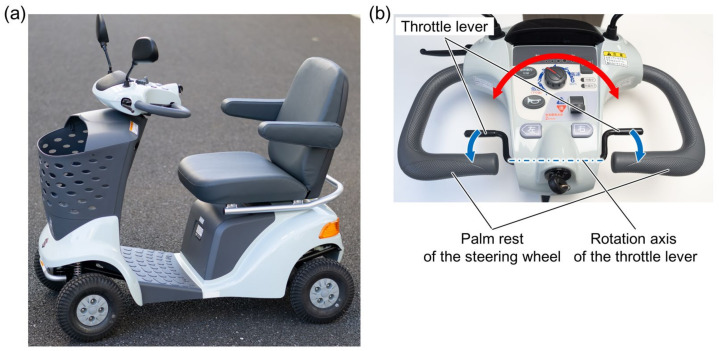
Motorized mobility scooter tested in this work. (**a**) Whole body structure. (**b**) Driving operation interfaces. The movable directions of the steering wheel and the throttle lever are indicated by the red and blue arrows, respectively.

Figure 1a shows the entire structure of the MMS (ET-4D; Suzuki Motor Corp., Shizuoka, Japan) that was used in this study for the concept validation. This test scooter has a steering wheel and throttle levers located in its front panel. The rotational axis of the steering wheel is connected to the front wheels via a mechanical linkage. As shown in Figure 1b, the right and left throttle levers have a common rotational axis that penetrates the front panel, and these levers are thus rotated with each other in an identical manner. Drivers usually place their palms on the body side bars of the steering wheel and push the throttle levers with their fingers. 

The main specifications of this mobility scooter are based on the appropriate Japan Industrial Standard (JIS T 9208:2016). The dimensions and weight of the scooter body are 1195 × 650 × 980 mm (length × width × height) and 100 kg, respectively. The upper driving speed limit can be set using the knob on the front panel, while the maximum vehicle speed is 6 km/h. During the test drive performed in this study, the limit was set at 4 km/h. The operable angle ranges of the steering wheel and the throttle levers were approximately ±50° and 40°, respectively.

### 2.2. Transformation of Gravitational Acceleration into the Operation Angles

The MMS requires specific structural characteristics to enable use of the IMUs for the monitoring of the steering wheel and throttle lever angles. First, when these operational interfaces have rotational axes and either one of them is not parallel with the gravitational vertical axis, both operational angles can be calculated from the gravitational acceleration measured by the IMUs attached to both the interfaces under a static condition. In addition, both angles can be calculated using a single IMU attached to the throttle lever if the two axes are not identical to each other. The test scooter described above has all these structural requisites. 

**Figure 2:**
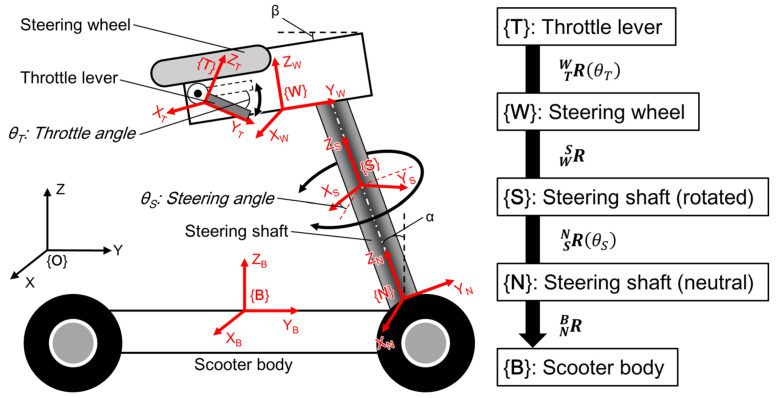
Coordinates and rotational order for the estimation of the operating angles (steering and throttle angles).

Figure 2 depicts the transformation scheme for the spatial coordinates from the coordinates along the scooter body {B} to the coordinates along the IMU attached to the throttle lever {T}. In a static state, the gravitational acceleration in {B} can be converted into that in {T} using four three-dimensional rotation matrices. These four matrices are as follows:

(1) RNB=1000cosα−sinα0sinαcosα

(2) RSN=cosθS−sinθS0sinθScosθS0001 

(3) RWS=1000cos−α−β−sin−α−β0sin−α−βcos−α−β 

(4) RTW=1000cos−θT−sin−θT0sin−θTcos−θT 

where *α* and *β* are the inclination angles of the steering shaft and the initial throttle lever position, respectively; and *θ_S_* and *θ_T_* are the steering and throttle angles, respectively. Using these matrices, the relationship between the three-axis acceleration measured by the IMU on the throttle lever and the gravitational acceleration in {B} can be expressed as follows:

(5) gB=RNBRSNRWSRTWaT

where aT is given as follows:

aT=aTxaTyaTzT

when the MMS is still on a flat surface, i.e., when {B} is identical to the absolute coordinate system {O}, gB can be assumed to be as follows:

gB=00gT

where *g* is the standard acceleration due to gravity (≈ 9.81 m/s^2^). However, the gravitational acceleration in {B} is altered by the inclination of the terrain and motion-induced inertia. To compensate for this acceleration change, it is necessary to use a second IMU attached to the scooter body. To perform this acceleration correction, gB is replaced with the three-axis outputs of the second IMU:

aB=aBxaByaBzT

and, by solving Equation (5), in which gB is replaced with aB, the steering and throttle angles can be obtained as follows:

(6) θS=sin−1aTxaBx2+C12−tan−1aBxC1

C1=aBycosα+aBzsinα

(7) θT=tan−1K2K1−sin−1aTyK12+K22−α−β

K1=−aBysinα+aBzcosαK2=−aBxsinθS+aBycosθScosα+aBzcosθSsinα

and, in the test MMS, the angles *α* and *β* were 20° and 9°, respectively.

### 2.3. Measurement Equipment for Accuracy Evaluation

**Figure 3:**
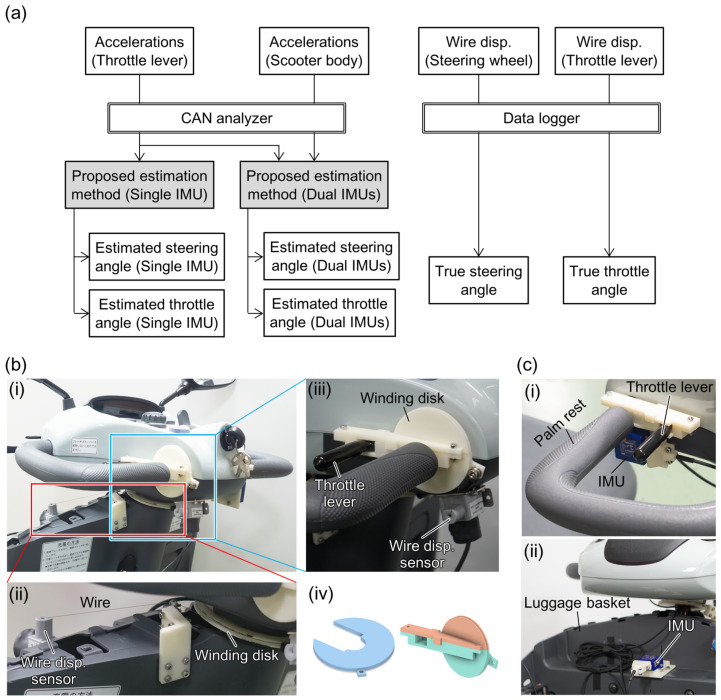
Measurement equipment used for the evaluation experiments. (**a**) The block diagram of the operating angle estimation and validation. (**b**) Measurement of the true values for the validation using wire displacement sensors. In (**i**) the control panel, the wire displacement sensors and the wire winding disks were mounted to measure the true (**ii**) steering and (**iii**) throttle angles. The designs for both winding disks are also shown in (**iv**). (**c**) IMUs used to estimate the operating angles. The IMUs mounted on (**i**) the throttle lever and (**ii**) the front luggage basket were used to perform angle calculations and compensate for the body inclination and movement, respectively.

To evaluate the accuracy of estimations of the operating angles when using the method proposed above, the test MMS was equipped with two different sensor types: IMUs for estimations using the proposed method and wire displacement sensors to measure the true operating angles of the steering wheel and the throttle lever. As shown in Figure 3a, the acceleration values of the IMUs attached to the throttle lever and the scooter body were used for the estimation of the operating angles (single IMU estimation) and acceleration compensation (dual IMUs estimation), respectively. The stored acceleration data were transformed into the operating angles using Equations (6) and (7) in the offline analysis.

Figure 3b,c show these sensors as they were installed on the scooter. The true operating angles were measured using wire displacement sensors (SM2, TE Connectivity Ltd., Schaffhausen, Switzerland), the wires of which were wound around circular disks that were attached to the rotational axes of both operational interfaces, as shown in Figure 3b. Using these structures, the rotation angles were transformed into the wire lengths wound by the disks. The two IMUs (LPMS CU2, LP-Research Inc., Tokyo, Japan) were attached to the throttle lever for the angle estimation and to the front luggage basket for the compensation of both movement- and inclination-induced acceleration, as shown in Figure 3c. The luggage basket is attached to the scooter body rather than to the panel of the steering wheel and is thus not rotated during turning operations. The winding disks and the attachment jigs for the IMUs were fabricated from acrylonitrile butadiene styrene using a fused deposition-modeling 3D printer.

The three-axis acceleration signals of the IMUs were digitized at 16 bit/100 Hz inside the units and recorded using a controlled area network analyzer (LE-270GR, Line Eye Co. Ltd., Kyoto, Japan). The voltage outputs of the wire displacement sensors were recorded using a versatile data logger (GL980, Graphtec Co. Ltd., Yokohama, Japan) at 100 Hz. Both timestamps were synchronized during the analysis process with the pulse signal from the Hall sensor that detected the approach of magnet chips attached to the rear wheel of the MMS for speed monitoring. 

### 2.4. Geometric Evaluation

**Figure 4:**
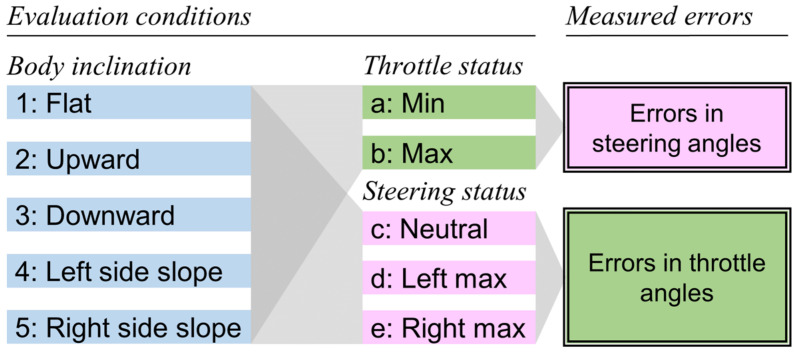
Evaluation conditions in the static state. Combinations of five conditions of the scooter’s body inclinations and two and three statuses of the throttle and the steering, respectively, were tested.

To confirm the geometric validity of the proposed method, the operating angle estimation errors were evaluated when the test scooter was in the static state. Figure 4 depicts the experimental conditions used to evaluate the estimation errors for the steering and throttle angles. The five types of scooter body inclination were combined with the two and three operating statuses of the throttle lever and the steering wheel, respectively. The five inclination conditions included a flat surface, upward and downward slopes with an angle of 5°, and left- and right-side slopes with an angle of 5°. 

Under each condition, either the steering wheel or the throttle lever was manually operated to move into seven or four different angular positions, respectively. Subsequently, the acceleration values obtained from the IMUs during each positioning operation were transformed into the operating angles using Equations (6) and (7). By subtracting a true angle from the estimated angles, the estimation error was obtained. A comparison of the errors among the conditions clarifies the extent to which the body inclination influences the estimation accuracy and to which the operating status of one interface influences the angle estimation of the other interface when using the proposed method.

### 2.5. Evaluation during Driving

**Figure 5:**
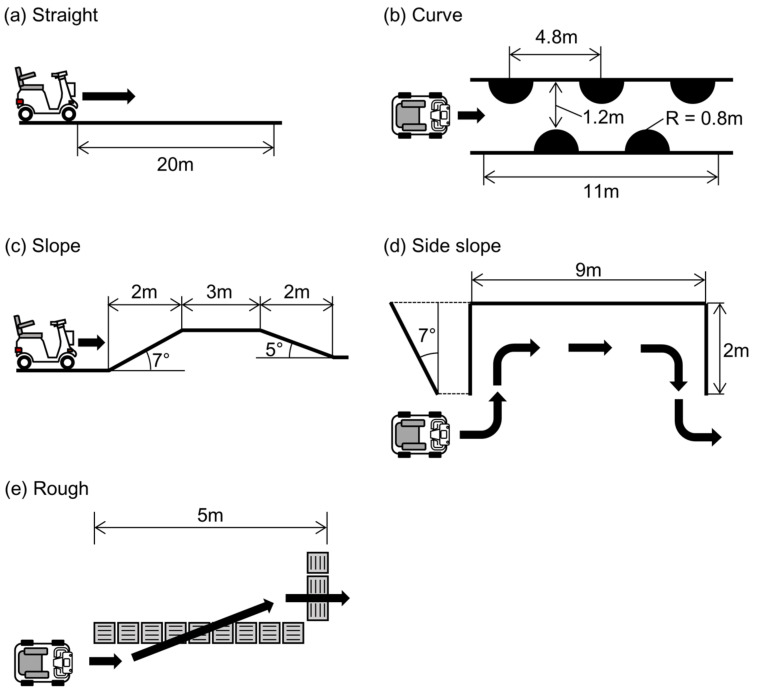
Test courses used for accuracy evaluation during driving. (**a**) Straight 20-m path. (**b**) Small curves. (**c**) Upward and downward slopes with angles of 7° and 5°, respectively. (**d**) Nine-meter side slope with an angle of 7°. (**e**) Path over a rough surface with tactile paving.

To evaluate the accuracy of the operating angle estimation when using the proposed method during driving, the test MMS was driven on the five different courses illustrated in Figure 5. The test courses included a 20-m-long straight path, small curves, upward and downward slopes with angles of 7° and 5°, respectively, a 9-m-long right-sided slope with an angle of 7°, and a course that crossed over a rough surface with tactile paving. Four healthy male participants (mean age (standard deviation, SD): 23.2 (0.5) year) drove the test scooter on these courses three times during the recording of the IMU signals and the true operating angles. The data from all participants were combined in the analysis.

To quantify the differences between the true and estimated values of the steering and throttle angles, the recorded data were processed using the following procedure. First, the data recorded during motion were extracted based on the signal from the Hall sensor that was used as a tachometer for the rear wheel. Then, the acceleration time series of the IMUs were smoothed using a 25th-order median filter to remove the spike noises caused by small vibrations. The filtered acceleration time series were transformed into angular values for the steering wheel and the throttle lever using the proposed method. To confirm the effectiveness of the compensation of both inclination- and motion-induced inertia with the IMU on the scooter body, the operating angles were estimated using both single and dual IMUs, and the results were compared with the true values. The data processing was performed using MATLAB (MathWorks Inc., Natick, MA, USA).

## 3. Results

### 3.1. Geometric Evaluation 

**Figure 6:**
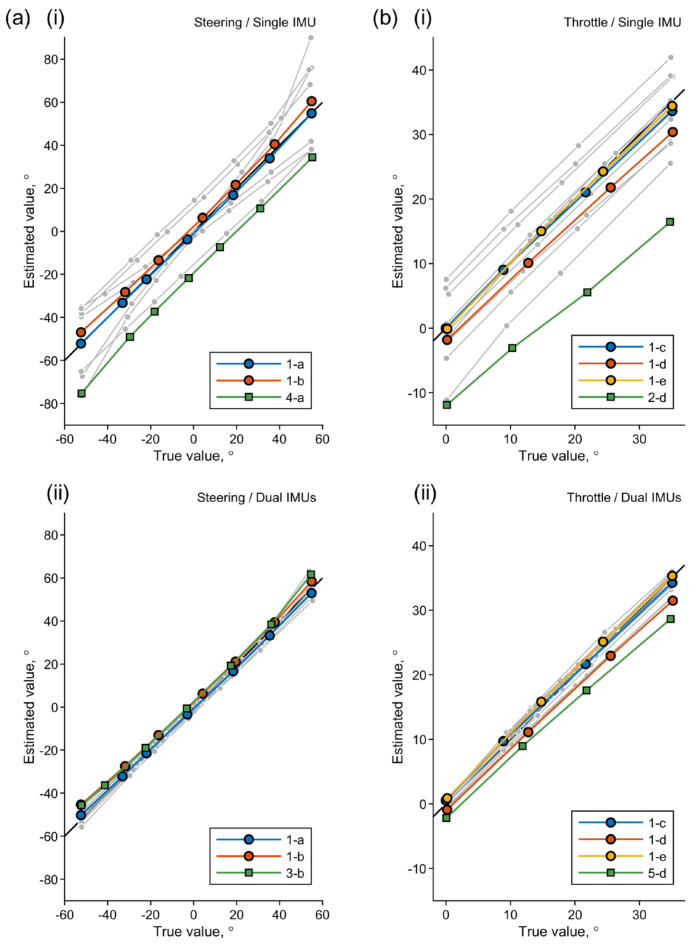
Estimated angles versus the true values from the static evaluation. (**a**) Steering and (**b**) throttle angles estimated with (**i**) a single IMU and (**ii**) dual IMUs are shown. The results for all the conditions given in Table 1 are plotted. The plots for the no inclination conditions and that with the maximum MAE for each panel are colored as shown in the legends, where the conditions are indicated using the column and row identifiers from Table 1.

Table 1 summarizes the results of the geometric evaluation, showing the mean absolute errors (MAEs) and SDs for 25 measurement conditions. The estimation errors acquired with a single IMU on the throttle lever and with an additional IMU located on the front basket for acceleration correction (dual IMUs) were compared to clarify the effects of the correction. Figure 6 shows line plots of the true and estimated angles for all the measurement and estimation conditions. For the sake of visibility, only the plots for the following conditions were colored: flat surface and neutral operation statuses (1-a and 1-b for the steering angle errors in (a); 1-c, 1-d, and 1-e for the throttle angle errors in (b)) and the conditions under which the maximum MAEs were observed in their respective panels.

Although the inclination of the scooter body caused large estimation errors when using a single IMU, the acceleration correction with use of the second IMU on the scooter body eliminated these errors successfully. While the MAEs with the single IMU exceeded 5° under 13 conditions, the corresponding errors with the dual IMUs remained under 5° under all conditions. This result clearly demonstrated that the geometric transformation described above was consistent with the structure of the test MMS. However, the operation of one interface affected the estimation accuracy for the other interface to some extent. The mechanical looseness or backlash in the rotational axes of both interfaces is one possible cause of this tendency [43]. The extent of the error, however, will be small enough to be negligible when the objective of the operational measurements is the monitoring of the driver behavior. 

### 3.2. Evaluation during Driving

**Figure 7:**
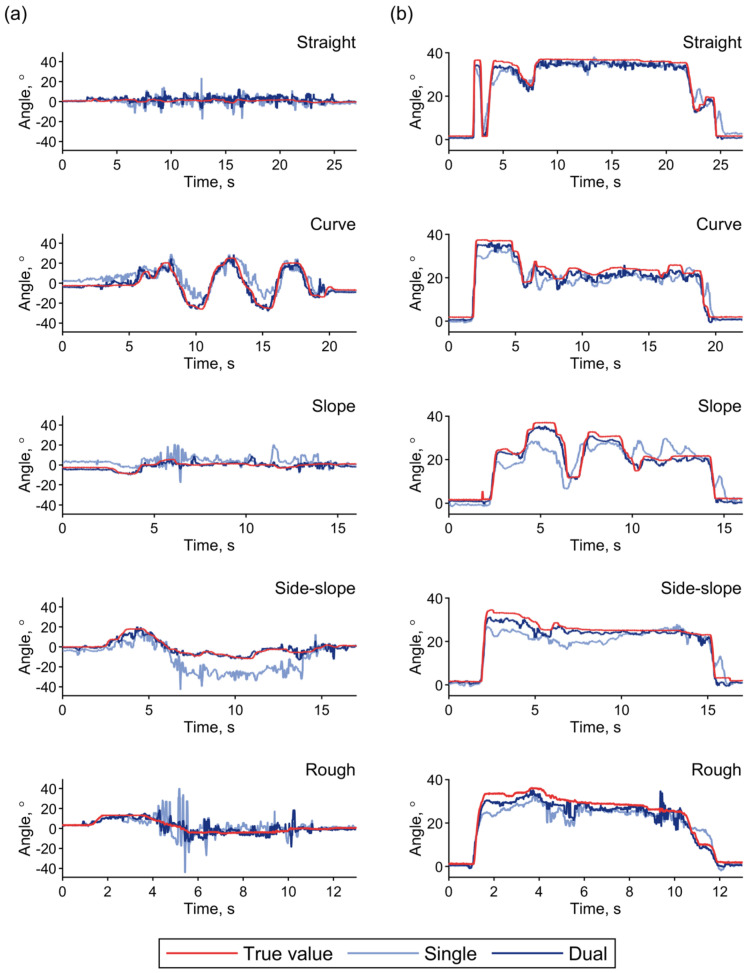
Typical time series values for the true and estimated operating angles during test driving over the five courses. (**a**) Steering angles. (**b**) Throttle angles. True values (red) are imposed on the estimated results obtained with the single (light blue) and dual (deep blue) IMUs.

Figure 7 shows the true and estimated time series values for the steering and throttle angles during the test driving over the five courses depicted in Figure 5. The effect of terrain inclination was clearly observed in the estimation with the single IMU during driving on the slope and side slope courses. As confirmed in the static state, the inclination-induced estimation errors were corrected in the estimations performed with dual IMUs. In addition to the inclination-induced error, the inertia during turning on the curved course caused a slight error in the estimation with the single IMU, which is obvious from the steering angle shown in Figure 7a. This error was also eliminated with the acceleration correction achieved through use of the additional IMU attached to the scooter body. 

**Figure 8:**
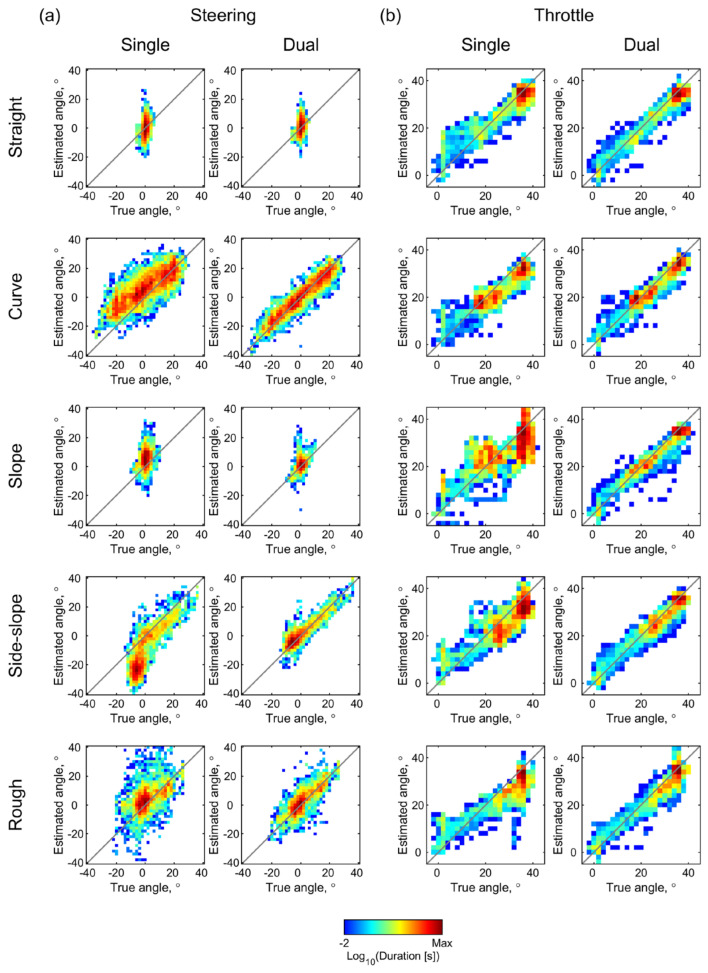
Estimated angles versus true angles during test driving over the five courses. The 2D histograms contain the recorded durations of the (**a**) steering and (**b**) throttle angles as estimated using single and dual IMUs in the pixels corresponding to the combinations of the true and estimated values. The recorded duration at each bin with widths of 2° in both axes is color-coded on the log scale.

The two-dimensional histograms of the estimation results shown in Figure 8 visualize these tendencies. Bins of the histogram with widths of 2° contain the measurement time durations that correspond to the respective combinations of the true and estimated angles, and they are color-coded on a logarithmic scale. When compared with the histograms for estimation with a single IMU in Figure 8a,b, the histograms for estimations with the dual IMUs demonstrated the acceleration correction effect across all the courses. Even in the straight course, a slight increase in the throttle angle that was estimated when using the single IMU could be observed in the lower range, as shown in Figure 8(bi), thus indicating the influence of the deceleration. This tendency is also observed in the time series plot of the throttle angle shown in Figure 7b and is reduced by the acceleration correction process with the dual IMUs.

**Figure 9:**
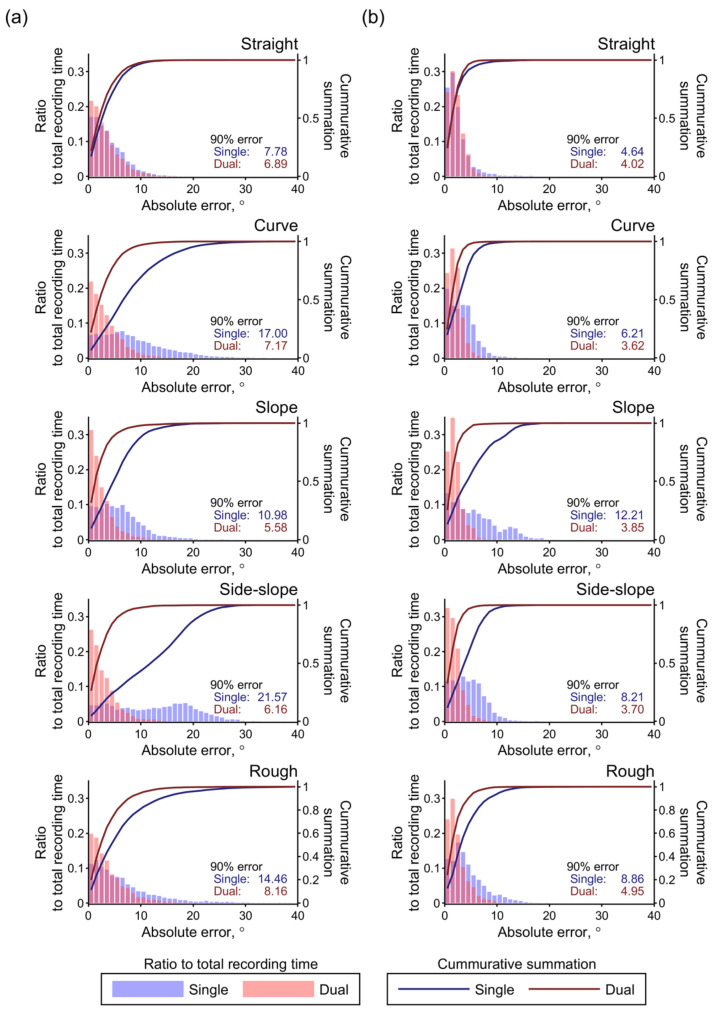
Distributions of the estimation errors obtained during test driving over the five courses. Histograms of the absolute errors and cumulative plots are shown for the (**a**) steering and (**b**) throttle angles. The results obtained with the single and dual IMUs are plotted in blue and red, respectively. The 90th percentile values of the error distributions are also shown in the panels.

Figure 9 shows histograms and 90th percentile values for the estimation errors for the five test courses and two estimation conditions. Over all the test courses, the 90% error values obtained with the dual IMUs were kept lower than those obtained with the single IMU through the acceleration correction effect. With the dual IMUs, the 90% errors in their entirety were lower than 10° and 5° for the steering and throttle angles, respectively. These values are approximately equal to 10% and 11.3% of the movable ranges for the two interfaces. 

Overall, the accuracy evaluation results obtained during driving demonstrated the feasibility of the proposed method. As a result of the acceleration correction performed using the IMU attached to the scooter body, the operating angles could be estimated with a practical accuracy level, even on terrains with inclinations and with inertia forces induced by the acceleration, deceleration, and turning behavior. 

## 4. Discussion

This study proposed and tested a novel IMU-based method to measure the driving operations of MMSs. The evaluation experiments demonstrated the feasibility of the proposed method to estimate the throttle and steering angles and quantified the estimation accuracies under various conditions. The geometric validations in the static state confirmed that these operating angles can be calculated from the acceleration values measured by the IMU attached to the throttle lever through a series of coordinate transformations, along with the mechanical rotation axes of the operating interfaces. The effects of terrain inclination can be compensated by use of the second IMU, which was attached to the scooter body. During driving, acceleration correction using the body IMU was also effective in reducing the inertia-induced estimation errors. 

To the best of the authors’ knowledge, this study represents the first attempt to quantify the driving operations of MMSs. As shown in previous studies on the measurements and analysis of PWC operations, precise quantification of the driving operation will provide insightful cues that will enable the characterization and improvement of the user driving styles [35,36,37,45]. Previous methods of skill evaluations for MMSs were mainly dependent on the rater’s observations of the driving performance [29]. Although such approaches are subject to the personal biases of the raters, it is practically acceptable that the evaluation procedure requires no special equipment. At this point, the proposed method is inferior to previous evaluation methods. However, the use of IMUs was proven to enable the implementation of the measurement system by simply attaching the sensors to the MMS without any modification of the internal electrical circuits. This feature of the proposed method will motivate further examination of the MMS operation logs in an effort to develop various applications in practical settings [37,45,46].

The important technical feature of the proposed method is that precise compensation of the terrain inclination and movement-induced inertia was achieved by solving the equation with the rotation matrices that represents the geometric structure of the MMS. The previous study that proposed an estimation method of a PWC joystick angle using IMUs adopted an approximate approach for the compensation, where the acceleration values of the IMU attached to the wheelchair body were simply subtracted from those of the IMU attached to the joystick [37]. This calculation naturally causes an estimation error due to the angular misalignment between the two IMU coordinates. The current method, therefore, has obvious merit in the estimation accuracy. This merit also leads to a reduction of the number of measurement parameters required for an estimation. The proposed method uses only acceleration values and does not require a complementary use of the angular velocities. Subsequently, the computing performance required for implementation of this method is considerably low. The data acquisition bitrate is at least 9.6 kbps for six 16-bit acceleration streams at 100 Hz, and this performance can be sufficiently achieved with low-cost single-board microcontrollers such as the Arduino series [47].

The proposed method also enabled the geometrical separation of the two operating angles when using a single IMU. If the measurements are performed on terrains without inclinations, the correction performed with the second IMU may not be necessary, depending on the accuracy required. This reduction in the number of sensing units required will be beneficial for practical use in the real-life environments of MMS users. For example, small smartphones such as Jelly Pro (Unihertz, Shanghai, China) can be used as replacements for the IMU to send the MMS operation logs directly to remote caregivers, including rehabilitation professionals, MMS providers, and care managers, who can then offer interventions to the users to improve their driving [48,49]. The previous investigations on MMS acquisition did not indicate that elderly MMS users would always have the required opportunities to obtain sufficient driving training [8,21,22]. Therefore, the easy-to-install and remotely operable monitoring system proposed here will have a considerable effect in promoting safe MMS use.

There have been no previous investigations of the permissible errors in the estimation of the MMS operating angles. A prior study of the angle estimation of a PWC joystick with IMUs reported a MAE that was under 10% of the maximum range [37]. This level of accuracy is approximately equivalent to that achieved using the current method. The required accuracy, however, is dependent on the objectives of the operational measurement and analysis. If necessary, it will be possible to improve the estimation accuracy by introducing a complementary or Kalman filter to fuse the accelerations and angular velocities into the operating angles [37,44,50]. The proposed method achieved a practical accuracy using only the accelerations from IMUs and did not require high hardware configurations for the use of the filter processing. Wu and colleagues, however, proposed a fast complementary filter for attitude estimations using magnetic, angular rate, and gravity sensor arrays, which successfully achieved a balance between the estimation accuracy and time consumption [50]. This approach will be suitable to implement real-time processing of the proposed method. The next issue to be addressed following this study will be to identify the information that can be extracted from an analysis of the operating angles of the MMSs and assess how this information can contribute to improvement of the user driving skills.

Despite the novelty and significance of the work discussed above, this study had a few limitations that must be noted. First, the proposed method was only tested on one type of MMS. The geometrical transformation of the measured accelerations into angles can amplify slight differences among the structural configurations, mechanical rigidities, and assembly accuracies of the MMSs. The estimation accuracy characteristic shown here can therefore be altered, depending on these factors. Second, the estimation results will also be affected by the terrain conditions [37]. The effects of the various terrain conditions that occur in real-life environments, which were not included in the test courses in this study, may require further examination. Third, the test measurement system consisted of off-the-shelf devices and may not be suitable for practical use. Optimization and customization of the entire system will still be necessary for practical applications.

## 5. Conclusions

This study proposed a novel IMU-based method to estimate the steering and throttle operating angles of MMSs, which can be simply implemented by attaching IMUs on the throttle lever and body of the MMS. The technical feature of the proposed method is the use of a series of coordinate transformations, along with the mechanical rotation axes of the operating interfaces. This precise modeling of the geometric structure of the MMS was effective in compensation of the terrain inclination and movement-induced inertia. The geometric validations in the static state provided proof of principle that these operating angles can be calculated through the proposed geometric transformations. The effects of terrain inclination were compensated by use of the second IMU, which was attached to the scooter body. During driving, acceleration correction using the body IMU was also effective in reducing the inertia-induced estimation errors. Overall, the 90% errors were lower than 10° and 5° for the steering and throttle angles, respectively, across all the terrain conditions tested. The proposed method will be used for quantitative and continuous monitoring of MMS driving behavior and act as a complementary tool for the existing skills measures for MMS driving that rely mostly on raters’ observations of task performances. 

## Figures and Tables

**Table 1:** Summary of the static evaluation of the estimation accuracy under the five inclination conditions (MAE (SD), °).

Body Inclination	Errors in Steering Angles		Errors in Throttle Angles		
	Throttle Position		Steering Position		
	a: Min	b: Max	c: Neutral	d: Left Max	e: Right Max
	**Single IMU**				
1: Flat	0.67 (0.61)	3.47 (1.48)	0.55 (0.66)	3.29 (1.23)	0.33 (0.24)
2: Upward	9.80 (9.30)	10.79 (12.07)	5.02 (0.60)	15.02 (2.85)	9.7 (1.04)
3: Downward	8.04 (5.69)	8.81 (5.65)	4.53 (0.51)	5.55 (0.98)	7.57 (0.40)
4: Left side slope	20.27 (1.42)	15.30 (1.75)	0.25 (0.30)	1.55 (0.75)	0.83 (0.74)
5: Right side slope	12.19 (1.43)	15.87 (2.59)	0.51 (0.41)	3.95 (1.72)	0.79 (0.44)
	**Dual IMUs**				
1: Flat	1.41 (0.76)	3.33 (1.95)	0.56 (0.38)	2.25 (1.15)	0.68 (0.34)
2: Upward	2.15 (1.56)	1.34 (1.16)	0.53 (0.20)	1.76 (0.96)	1.00 (0.57)
3: Downward	1.45 (0.71)	4.10 (2.09)	0.29 (0.36)	1.55 (1.16)	0.81 (0.36)
4: Left side slope	3.57 (1.30)	2.27 (1.59)	0.49 (0.30)	0.82 (0.68)	1.23 (0.74)
5: Right side slope	1.18 (0.68)	3.25 (2.82)	0.51 (0.46)	3.90 (1.73)	0.79 (0.42)

## Data Availability

The data that support the findings of this study are available upon reasonable request to the corresponding author.

## References

[1] Fomiatti R., Richmond J., Moir L., Millsteed J. (2013). A systematic review of the impact of powered mobility devices on older adults’ activity engagement. Phys. Occup. Ther. Geriatr..

[2] Thoreau R. (2015). The impact of mobility scooters on their users. Does their usage help or hinder?: A state of the art review. J. Transp. Health.

[3] Frank A., Neophytou C., Frank J., De Souza L. (2010). Electric-powered indoor/outdoor wheelchairs (EPIOCs): Users’ views of influence on family, friends and carers. Disabil. Rehabil. Assist. Technol..

[4] Ripat J., Verdonck M., Carter R.J. (2018). The meaning ascribed to wheeled mobility devices by individuals who use wheelchairs and scooters: A metasynthesis. Disabil. Rehabil. Assist. Technol..

[5] Fredriksson C., Pettersson I., Hagberg L., Hermansson L. (2020). The value of powered mobility scooters from the perspective of elderly spouses of the users–a qualitative study. Disabil. Rehabil. Assist. Technol..

[6] Löfqvist C., Pettersson C., Iwarsson S., Brandt A. (2012). Mobility and mobility-related participation outcomes of powered wheelchair and scooter interventions after 4-months and 1-year use. Disabil. Rehabil. Assist. Technol..

[7] Sund T., Brandt Å. (2018). Adult Scandinavians’ use of powered scooters: User satisfaction, frequency of use, and prediction of daily use. Disabil. Rehabil. Assist. Technol..

[8] Mortenson W.B., Kim J. (2016). Scoping review of mobility scooter-related research studies. J. Rehabil. Res. Dev..

[9] Jancey J., Cooper L., Howat P., Meuleners L., Sleet D., Baldwin G. (2013). Pedestrian and Motorized Mobility Scooter Safety of Older People. Traffic Inj. Prev..

[10] Kitching F.A., Ozanne-Smith J., Gibson K., Clapperton A., Cassell E. (2016). Deaths of older Australians related to their use of motorised mobility scooters. Int. J. Inj. Contr. Saf. Promot..

[11] Murphy C.G., Murphy I.G., O’Rourke K.S., O’Shea K. (2014). Motorised mobility scooters; upper limb fractures in elderly novice users. Clin. Cases Miner. Bone Metab..

[12] Gitelman V., Pesahov F., Carmel R., Chen S. (2017). Exploring the characteristics of potential and current users of mobility scooters, among older people in Israel. Transp. Res. Part F Traffic Psychol. Behav..

[13] Isaacson M., Barkay D. (2020). Mobility scooters in urban environments: A research agenda. J. Transp. Health.

[14] Henje C., Stenberg G., Lundälv J., Carlsson A. (2021). Obstacles and risks in the traffic environment for users of powered wheelchairs in Sweden. Accid. Anal. Prev..

[15] Akter S., Mamun M.M.H., Mwakalonge J.L., Comert G., Siuhi S. (2021). A policy review of electric personal assistive mobility devices. Transp. Res. Interdiscip. Perspect..

[16] Mortenson W.B., Clarke L.H., Best K. (2013). Prescribers’ experiences with powered mobility prescription among older adults. Am. J. Occup. Ther..

[17] Pellichero A., Kenyon L.K., Best K.L., Sorita É., Lamontagne M.E., Lavoie M.D., Routhier F. (2020). Influence of cognitive functioning on powered mobility device use: Protocol for a systematic review. JMIR Res. Protoc..

[18] Suzurikawa J., Sawada Y., Sakiyama M., Suwa M., Inoue T., Kondo T. (2021). Perspectives of Multidisciplinary Professional Teams during Assessment Processes for ATD Selection in the Japanese Public Provision System. Int. J. Environ. Res. Public Health.

[19] Edwards K., McCluskey A. (2010). A survey of adult power wheelchair and scooter users. Disabil. Rehabil. Assist. Technol..

[20] Sullivan S.J., La Grow S., Alla S., Schneiders A.G. (2014). Riding into the future: A snapshot of elderly mobility scooter riders and how they use their scooters. N. Z. Med. J..

[21] Widehammar C., Lidström Holmqvist K., Pettersson I., Hermansson L.N. (2020). Attitudes is the most important environmental factor for use of powered mobility devices–users’ perspectives. Scand. J. Occup. Ther..

[22] Pettersson C., Iwarsson S., Brandt Å., Norin L., Lexell E.M. (2014). Men’s and women’s perspectives on using a powered mobility device: Benefits and societal challenges. Scand. J. Occup. Ther..

[23] Carlsson A., Lundälv J. (2019). Acute injuries resulting from accidents involving powered mobility devices (PMDs)—Development and outcomes of PMD-related accidents in Sweden. Traffic Inj. Prev..

[24] Eck D., Schilling K., Abdul-Majeed A., Thielecke J., Richter P., Boronat J.G., Schens I., Thomas B., Williger B., Lang F.R. (2012). Mobility assistance for older people. Appl. Bionics Biomech..

[25] Liu K., Mulky R. (2018). Enabling autonomous navigation for affordable scooters. Sensors.

[26] Cecotti M., Kanchwala H., Aouf N. Autonomous Navigation for Mobility Scooters: A Complete Framework Based on Open-Source Software. Proceedings of the 2019 IEEE Intelligent Transportation Systems Conference (ITSC).

[27] Thoreau R. (2019). Perception of needing and using a mobility scooter: A preclinically disabled non-scooter user perspective. Disabil. Rehabil. Assist. Technol..

[28] Nitz J.C. (2008). Evidence from a cohort of able bodied adults to support the need for driver training for motorized scooters before community participation. Patient Educ. Couns..

[29] Toosizadeh N., Bunting M., Howe C., Mohler J., Sprinkle J., Najafi B. (2014). Motorized mobility scooters: The use of training/intervention and technology for improving driving skills in aging adults—A mini-review. Gerontology.

[30] Mortenson W.B., Jang S., Goldsmith C.H., Hurd Clarke L., Hobson S., Emery R. (2017). Feasibility of a Systematic, Comprehensive, One-to-One Training (SCOOT) program for new scooter users: Study protocol for a randomized control trial. Trials.

[31] Dawson D., Chan R., Kaiserman E. (1994). Development of the Power-Mobility Indoor Driving Assessment for Residents of Long-Term Care Facilities: A Preliminary Report. Can. J. Occup. Ther..

[32] Letts L., Dawson D., Kaiserman-Goldenstein E. (1998). Development of the Power-mobility Community Driving Assessment. Can. J. Rehabil..

[33] Mountain A.D., Kirby R.L., Smith C. (2004). The wheelchair skills test, version 2.4: Validity of an algorithm-based questionnaire version. Arch. Phys. Med. Rehabil..

[34] Routhier F., Vincent C., Desrosiers J., Nadeau S., Guerette C. (2004). Development of an obstacle course assessment of wheelchair user performance (OCAWUP): A content validity study. Technol. Disabil..

[35] Suzurikawa J., Kinoshita T., Inoue T., Kamo M., Iida N., Iwata K., Matsumoto O. (2012). Evaluation of changes in power wheelchair maneuver induced by a downhill turning prevention control on cross sloped surfaces. IEEJ Trans. Electr. Electron. Eng..

[36] Sorrento G.U., Archambault P.S., Routhier F., Dessureault D., Boissy P. (2011). Assessment of Joystick control during the performance of powered wheelchair driving tasks. J. Neuroeng. Rehabil..

[37] Rabreau O., Chevallier S., Chassagne L., Monacelli E. (2019). SenseJoy, a pluggable solution for assessing user behavior during powered wheelchair driving tasks. J. Neuroeng. Rehabil..

[38] Jannink M.J.A., Erren-Wolters C.V., De Kort A.C., Van Der Kooij H. (2008). An electric scooter simulation program for training the driving skills of stroke patients with mobility problems: A pilot study. Cyberpsychol. Behav..

[39] Cordes C., Heutink J., Brookhuis K.A., Brouwer W.H., Melis-Dankers B.J.M. (2018). Driving slow motorised vehicles with visual impairment—A simulator study. Cogent Psychol..

[40] Heutink J., Broekman M., Brookhuis K.A., Melis-Dankers B.J.M., Cordes C. (2019). The effects of habituation and adding a rest-frame on experienced simulator sickness in an advanced mobility scooter driving simulator. Ergonomics.

[41] Seel T., Raisch J., Schauer T. (2014). IMU-based joint angle measurement for gait analysis. Sensors.

[42] Fasel B., Spörri J., Schütz P., Lorenzetti S., Aminian K. (2017). Validation of functional calibration and strap-down joint drift correction for computing 3D joint angles of knee, hip, and trunk in alpine skiing. PLoS ONE.

[43] Vitali R.V., Perkins N.C. (2020). Determining anatomical frames via inertial motion capture: A survey of methods. J. Biomech..

[44] Potter M.V., Cain S.M., Ojeda L.V., Gurchiek R.D., McGinnis R.S., Perkins N.C. (2021). Error-state Kalman filter for lower-limb kinematic estimation: Evaluation on a 3-body model. PLoS ONE.

[45] Routhier F., Lettre J., Miller W.C., Borisoff J.F., Keetch K., Mitchell I.M. (2019). Data Logger Technologies for Powered Wheelchairs: A Scoping Review. Assist. Technol..

[46] Pineau J., Moghaddam A.K., Yuen H.K., Archambault P.S., Routhier F., Michaud F., Boissy P. (2014). Automatic detection and classification of unsafe events during power wheelchair use. IEEE J. Transl. Eng. Health Med..

[47] Haghi M., Thurow K., Stoll R. (2017). Wearable devices in medical internet of things: Scientific research and commercially available devices. Healthc. Inform. Res..

[48] Kim W., Lee S., Kim S., Jo S., Yoo C., Hwang I., Kang S., Song J. (2020). Dyadic Mirror: Everyday Second-person Live-view for Empathetic Reflection upon Parent-child Interaction. Proc. ACM Interact. Mob. Wearable Ubiquitous Technol..

[49] Kao H.T., Yan S., Hosseinmardi H., Narayanan S., Lerman K., Ferrara E. (2020). User-Based Collaborative Filtering Mobile Health System. Proc. ACM Interact. Mob. Wearable Ubiquitous Technol..

[50] Wu J., Zhou Z., Chen J., Fourati H., Li R. (2016). Fast Complementary Filter for Attitude Estimation Using Low-Cost MARG Sensors. IEEE Sens. J..

